# Influence of gestational diabetes mellitus on lipid signatures in breast milk and association with fetal physical development

**DOI:** 10.3389/fnut.2022.924301

**Published:** 2022-08-10

**Authors:** Hong Zhong, Jiahua Zhang, Jiaai Xia, Yuting Zhu, Chen Chen, Chunjian Shan, Xianwei Cui

**Affiliations:** ^1^School of Nursing, Nanjing Medical University, Nanjing, China; ^2^Nanjing Maternal and Child Health Institute, Women’s Hospital of Nanjing Medical University, Nanjing Maternity and Child Health Care Hospital, Nanjing, China; ^3^Department of Obstetrics and Gynecology, Women’s Hospital of Nanjing Medical University, Nanjing Maternity and Child Health Care Hospital, Nanjing, China

**Keywords:** gestational diabetes mellitus, breastfeeding, lipidomics, maternal glucose, infant physical development

## Abstract

Gestational diabetes mellitus (GDM) commonly leads to adverse pregnancy outcomes and long-term metabolic complications in offspring. Breastfeeding has been shown to rewrite the fetal “metabolic programming” resulting from maternal diabetes and finally lead to a lower risk of future metabolic disease. Lipids in breast milk act like hormones to promote infant growth and development, but there is minimal information invested thus far in constitution changes of lipids in breast milk, especially in the context of GDM. In the present study, we performed a lipidomics analysis to compare the lipid composition in breast milk collected from women with or without GDM. We further revealed the correlations of dysregulated lipids in breast milk with maternal glucose and infant physical development. A total of 833 lipid species from 15 classes were identified, 60 of which were found to be significantly altered in response to the high glucose, suggesting a remarkable lipid profiling change in breast milk induced by GDM. Our results showed significant associations between dysregulated lipids (e.g., neutral lipids, phospholipids, sphingolipids) and maternal glucose. Furthermore, correction analysis demonstrated that GDM related lipids were also associated with indicators of infant physical development, including body weight, length, and head circumference. These findings may help to understand the protective effects of breastfeeding especially during GDM pregnancy.

## Introduction

Gestational diabetes mellitus (GDM) is diagnosed by the first recognition or onset of hyperglycemia or impaired glucose tolerance during pregnancy (1). With the improvement of living standards, delayed gestational age, excess nutrition and lack of exercise, GDM has increased considerably and become a dominant complication affecting 9.3–25.5% of pregnancies globally (2). In addition to serious maternal illnesses, including shoulder dystocia, pregnancy-induced hypertension, and cesarean section, infants born to mothers with GDM are at high risk for obesity and even type 2 diabetes (T2D) when growing up (3). These youth who have been exposed to maternal diabetes have been “programmed” *in utero* and thus are more prone to develop metabolic disorders later in life when exposed to excess fuels such as glucose (4). It was reported that postnatal growth environments, especially early nutrition, play a critical role in shaping the growth and development of the neonate (5). Thus, appropriate nutritional intervention may help to correct the programmed development and reduce the future metabolic complications in the offspring of women with GDM.

Breast milk is the perfectly adapted personalized nutritional supply for infants. In addition to providing all the elementary nutrients for a baby’s growth and development, breast milk feeding confers multiple protection against infection and inflammation and contributes to immune maturation, initial microbial colonization and organ development (6). Notably, long-lasting effects from breastfeeding, such as a lower risk of overweight, obesity, and development of T2D in mid of later life, have also been demonstrated (7). There is growing evidence that breastfeeding has health benefits for both mothers with GDM and their infants. Breastfeeding improves women’s lipid and glucose metabolic profiles with a history of GDM immediately after delivery, and reduces the risk of progression to T2D later in life (8). Breastfeeding for at least 6 months reduced childhood fat deposition resulting from exposure to GDM *in utero* (9). Components (i.e., proteins, lipids, and carbohydrates) of human milk may rewrite the fetal “metabolic programming” induced by GDM in the early postpartum period, leading to a lower risk of future metabolic disease (10–12). A recent study revealed that the breastmilk microbiota composition was also influenced by GDM and these changes may contribute to the dynamics of the infants’ gut microbiota establishment (13). Therefore, it was interesting to explore which components in human milk shed protective effects on the growth and development of infants.

Besides supplying the baby’s caloric intake (45–55%), breast milk lipids also perform numerous functions in regulating infants’ short- and long-term health (14). The major composition of breast milk fat is triacylglycerols (TAGs), representing 98% of total lipids, while other lipid species like phospholipid (PL), diacylglycerol (DAG), monoacylglycerol (MAG), and sphingomyelin (SM) are also detected, but with different abundance (15). Increasing attention has been focused on the biological effects of human milk lipids, including anti-inflammatory, neurocognitive development, prebiotic functions, etc. (16). Lipids in breast milk also have been reported to act like hormones to promote infant growth and development. For example, 12,13-diHOME, a metabolite in breast milk boosted after maternal exercise, was said to benefit offspring in terms of infant weight gain and body adiposity in the early postnatal period (17). Breast milk contains more than 400 different lipid species and could evolve to meet the unique requirements of the infants (18, 19). However, there is minimal information invested thus far in lipid profile of breast milk, especially in the context of GDM.

In the present study, we performed mass spectrometry-based lipidomics analysis to compare the lipid composition in colostrum collected from women with or without GDM pregnancy. A total of 833 lipids from 15 classes were identified, 60 of which were found to be significantly dysregulated resulting from the maternal diabetic state. We then linked these lipids to maternal glucose and found a remarkable association with decreased neutral lipids and increased PLs/sphingolipids. Finally, we correlated the lipids related to GDM in breast milk with infant physical development.

## Materials and methods

### Sample collection

Milk samples were obtained from women who delivered at Nanjing Maternity and Child Health Care Hospital from September 2021 to December 2021. Milk was collected through an electronic breast pump within 3 days after birth and stored at −80°C until processed. The project was approved by the Human Research Ethics Committee of the Hospital with a permit number of 2020KY075. A total of 40 pregnant women was enrolled in this study, including 20 GDM women and 20 matched controls. Women with GDM were screened out when undergoing the routine 75-g oral glucose tolerance test (OGTT) at 24–28 weeks of pregnancy. Specifically, a cut-off of fasting glucose levels ≥ 5.10 mmol/L, 1-h glucose levels ≥ 10.00 mmol/L, or 2-h glucose levels ≥ 8.50 mmol/L in OGTT was confirmed for GDM. This study excluded pregnant women who were also complicated by diabetes, chronic hypertension, thyroid disease, or autoimmune disease.

### Lipids extraction

A 100 μL aliquot of colostrum was mixed with 480 μL of extract solution (methyl tertiary butyl ether: methanol = 5:1) and sonicated for 10 min in an ice-water bath. The mixed samples were incubated at −40°C for 1 h and centrifuged at 4°C, 3000 rpm for 15 min. 350 μL of supernatant was collected and dried in a vacuum concentrator. The dried samples were further reconstituted in 100 μL of 50% methanol in dichloromethane by sonication for 10 min in an ice-water bath. The constitution was then centrifuged at 4°C, 13000 rpm for 15 min, and 75 μL of supernatant was transferred to a fresh glass vial for liquid chromatography-tandem mass spectrometry (LC-MS). The quality control (QC) samples were randomly injected for every 10 experimental samples to assess the stability of the LC–MS.

### LC-MS/MS analyses

Untargeted LC-MS analyses were performed using an Ultra-High-Performance Liquid Tandem Chromatography Quadrupole Time of Flight Mass Spectrometry (UHPLC-QTOFMS, 1290, Agilent Technologies) by Biotree Biomedical Technology Company in Shanghai. Lipid species separation was conducted on a Kinetex C18 column (2.1 × 100 mm, 1.7 μm, Phenomen) after equilibrating. The mobile phase comprised component A (0 mmol/L ammonium formate, 60% acetonitrile solution, and 40% water) and component B (10% acetonitrile together with 90% isopropanol), respectively. The gradient elution program was initiated with 40% eluent B, which was ramped to 100% from 0 to 10 min, and the gradient held constant at 100% B from 12.0 to 13.5 min, after which the mobile phase was decreased from 100 to 40% B from 13.5 to 13.7 min, followed by equilibration of the column at 40% B from 13.7 to 18.0 min. An injection volume of 2 μL for both positive and negative models was used. Lipid extracts were injected randomly using one QC sample injected every ten real samples for stability LC–MS control. MS/MS spectra were obtained from the Q Exactive Orbitrap Mass Spectrometer on data-dependent acquisition mode, following the previous strategy. The ESI source conditions were operated under the following conditions: the capillary voltages were 5000 and −4500 V, the capillary temperatures were 320 and 300°C, and the sheath gas and Aux gas flow rates were 30 and 10 Arb, in positive mode and in negative mode, respectively. The full MS resolution was 70000, the MS/MS resolution was 17500, and the collision energy was 15/30/45 in NCE mode.

### Data processing

The collected peaks were filtered deviation values based on the coefficient of variation and normalized by the internal standard. Preprocessed data were identified using ProteoWizard’s msconvert program, and further lipid identification was achieved through a spectral match using the LipidBlast library. Standardized data containing the information of peak number, sample name, and normalized peak area was extracted from the SIMCA16.0.2 software package (Sartorius Stedim Data Analytics AB, Umea, Sweden). Principal component analysis (PCA) was performed to distinguish and group the samples, and the orthogonal projections to latent structures-discriminant analysis (OPLS-DA) was further analyzed to find significantly changed lipids. Furthermore, the value of variable importance in the projection (VIP) of the first principal component in OPLS-DA analysis was acquired to summarize the contribution of each variable to the model. The lipids with VIP more than 1 and *P* < 0.05 (Student’s *t*-test) were considered statistically significant.

### Statistical analysis

The clinical data from maternal and offspring are shown as mean ± standard deviation (SD). The distribution, fold change and structure of identified lipids were performed using GraphPad Prism 7.0 (San Diego, CA, United States). Correlation analysis between GDM related lipids and clinical data was done in IBM SPSS Statistics 26.0. The variations in different groups were analyzed using the Student’s *t*-tests, and *P*-value < 0.05 was considered statistically significant.

## Results

### Clinical characteristics of participants

The basic information of 40 pregnant women recruited in the present study was summarized in Table 1. There was no significant difference in maternal age and gestational age between women who developed GDM and those who did not (*P* > 0.05). The pre-pregnancy and third trimester body mass index (BMI) of the mothers were comparable between these two groups (Supplementary Table 1). As expected, the fasting plasma glucose (FPG), 1 h plasma glucose (1hPG), and 2 h plasma glucose (2hPG) from OGTT were much higher in the GDM group when compared to normal controls (NC) (*P* > 0.001). We next investigated the concentration changes of serum lipids, including TG (triglyceride), TC (total cholesterol), LDLC (low-density lipoprotein cholesterol), and HDLC (high-density lipoprotein cholesterol). Of note, higher TG level was observed in GDM group (*P* < 0.05), whereas the concentrations of TC, LDLC, and HDLC did not show any changes between these two groups.

**TABLE 1:** Clinical characteristics of pregnant women with or without GDM.

	NC (*n* = 20)	GDM (*n* = 20)	*P*-value
Maternal age (years)	30.7 ± 1.92	29.6 ± 2.87	0.16
BMI (kg/m^2^)	26.31 ± 2.20	27.38 ± 3.12	0.22
Gestational age (weeks)	38.8 ± 0.83	39.05 ± 0.83	0.35
FPG (mmol/L)	4.31 ± 0.28	5.00 ± 0.48	<0.001**
1hPG (mmol/L)	7.41 ± 1.46	9.52 ± 1.06	<0.001**
2hPG (mmol/L)	6.33 ± 1.19	7.79 ± 1.45	<0.001**
TG (mmol/L)	2.78 ± 0.87	3.74 ± 1.57	0.02*
TC (mmol/L)	6.66 ± 0.93	6.94 ± 1.25	0.41
LDLC (mmol/L)	3.52 ± 0.78	4.02 ± 1.07	0.10
HDLC (mmol/L)	2.13 ± 0.96	2.43 ± 0.35	0.19

Data are shown as mean ± SD. * P < 0.05, **P < 0.01 vs. NC group, unpaired t-test. The bold values mean significant difference. SD, standard deviation; GDM, Gestational diabetes mellitus; NC, normal controls; FPG, fasting plasma glucose; 1hPG, 1 h plasma glucose; 2hPG, 2 h plasma glucose; TG, triglyceride; TC, total cholesterol; LDLC, low density lipoprotein cholesterol; HDLC, high density lipoprotein cholesterol.

### Lipid profiling changes in breast milk response to gestational diabetes mellitus

**FIGURE 1:**
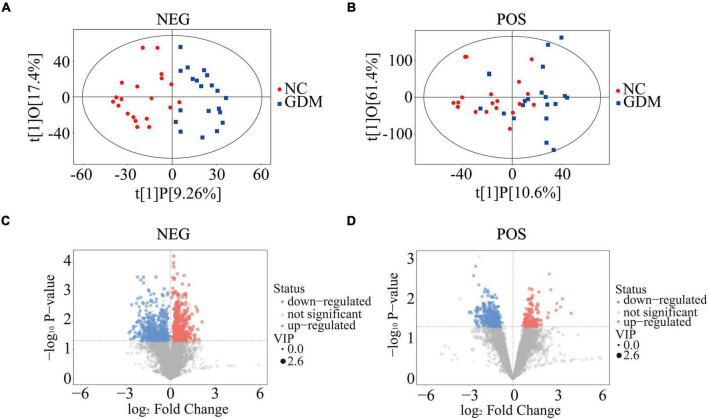
Lipid profiling changes in breast milk response to GDM. Score scatter plots of OPLS-DA for negative ion mode **(A)** and positive ion mode **(B)**. Volcano plots showed the difference of molecular features in negative ion mode **(C)** and positive ion mode **(D)**. OPLS-DA, orthogonal projections to latent structures-discriminate analysis.

To explore changes in lipid composition of breast milk affected by maternal diabetic state, we performed a broad spectrum lipidomics analysis using colostrum samples from 40 pregnant women with or without GDM. After relative standard deviation de-noising, 7,575 peaks for negative ion mode and 9,015 peaks for positive ion mode were obtained. PCA and OPLS-DA were then used to visualize the separability among the collected breast milk samples based on the lipid pattern. Although PCA indicated partial separability of lipid profiles between GDM and NC groups (Supplementary Figures 1A,B), the score scatter plots of the OPLS-DA model suggested that the two groups of samples could be almost wholly separated within the 95% confidence interval (Figures 1A,B). OPLS-DA can filter out the orthogonal variables in the lipids that were not related to the classification variables, so as to better show the differences of lipid profile between GDM group and NC group. Moreover, the displacement test of OPLS-DA model showed that the original model had good robustness and there was no obvious over-fitting phenomenon (Supplementary Figures 1C,D). To further indicate the differentially expressed molecular features between GDM group and NC group, the VIP values calculated in the OPLS-DA model greater than 1 and *P*-values of less than 0.05 (Student’s *t*-test) have been applied for statistical significance. All different molecular features were summarized and shown in the volcanic map (Figures 1C,D). Specifically, 981 molecular features in negative ion mode and 400 in positive ion mode were picked out between GDM group and NC group. Detailed information about differently expressed features was shown in Supplementary Tables 2, 3. All these results indicated that the lipid composition in colostrum is significantly changed in response to GDM pregnancy.

### Classification of breast milk lipids linking to gestational diabetes mellitus

**FIGURE 2:**
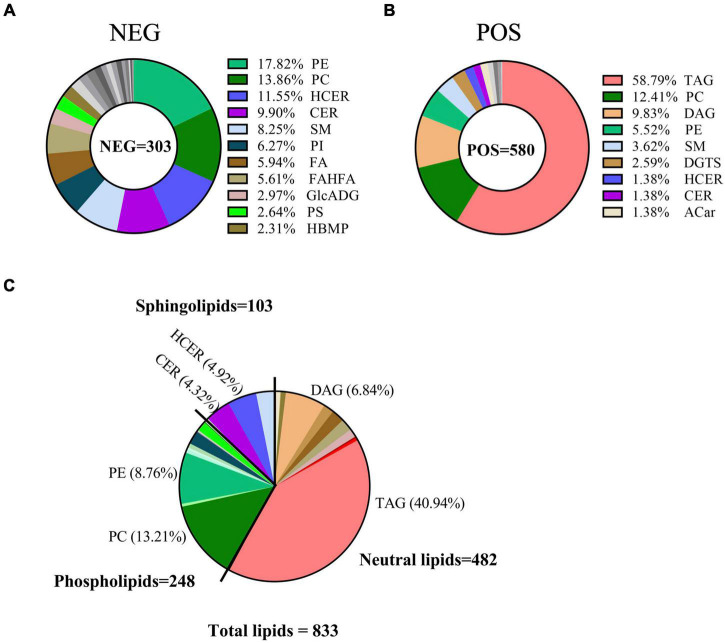
Classification of breast milk lipids linking to GDM. Pie charts showed the classification of lipids in breast milk relating to GDM in negative ion mode **(A)**, positive ion mode **(B)**, and total identified lipids **(C)**, respectively.

Next, we matched all molecular features to the LipidBlast library. A total of 833 lipids were identified for further analysis in breast milk, including 303 lipids in negative ion mode and 580 lipids in positive ion mode (Supplementary Tables 4, 5). Of the 303 lipids in negative ion mode, 17.82% were from phosphatidylethanolamine (PE), 13.86% from phosphatidylcholine (PC), 11.55% from hexosylceramide (HCER), 9.9% from Ceramide (CER), 8.25% from SM, 6.27% from phosphatidylinositol (PI), 5.94% from fatty acids (FA), and so on (Figure 2A). In contrast, of the 580 lipid species in positive ion mode, 58.79% were TAG, 12.41% were PC, 9.83% were DAG, 5.52% were PE, and 3.62% were SM (Figure 2B). When merging lipids from these two modes together, the following classes were represented: neutral lipids class (like TAG and DAG, 482 lipid species), PLs class (like PC and PE, 248 lipid species), and sphingolipids class (like HCER and CER, 103 lipid species) (Figure 2C).

### Lipids changes in breast milk correlated with maternal glucose

**FIGURE 3:**
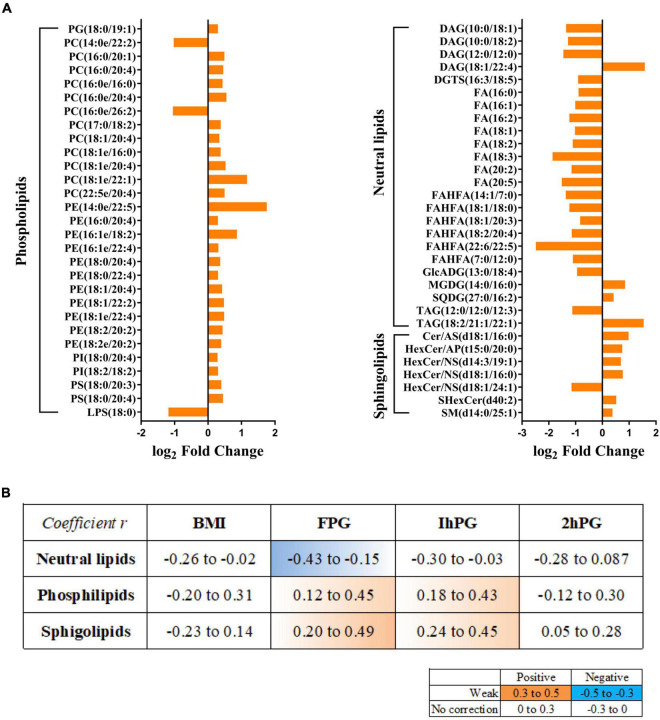
Lipids changes in breast milk correlate with maternal glucose. **(A)** Fold changes of significantly altered lipids between GDM group and NC groups. **(B)** Correlations between GDM related lipids and maternal clinical parameters. Orange color indicates a positive correlation, and blue exhibits negative correction.

In particular, of the 833 lipid species, 60 of which were significantly altered between GDM group and NC group, containing 36 up-regulated lipids and 24 down-regulated lipids (Supplementary Table 6; Figure 3A). The majority of up-regulated lipids were clustered into the PLs and sphingolipids, whereas the down-regulated lipids were mainly summarized as neutral lipids. The most abundant breast milk lipid in GDM group was PE (14:0e/22:5), followed by PC (18:1e/22:1). Conversely, FAHFA (22:6/22:5), a differential neutral lipid, was the most abundant breast milk lipid in NC group. Then we linked these lipid species to the clinical parameters of participants, such as BMI, FPG, 1hPG, and 2hPG. Notably, neutral lipids were shown to have a negative correlation with FPG, while PLs and sphingolipids demonstrated positive correlations with FPG and 1hPG (Figure 3B).

### Breast milk lipids structure analysis in relation to gestational diabetes mellitus

**FIGURE 4:**
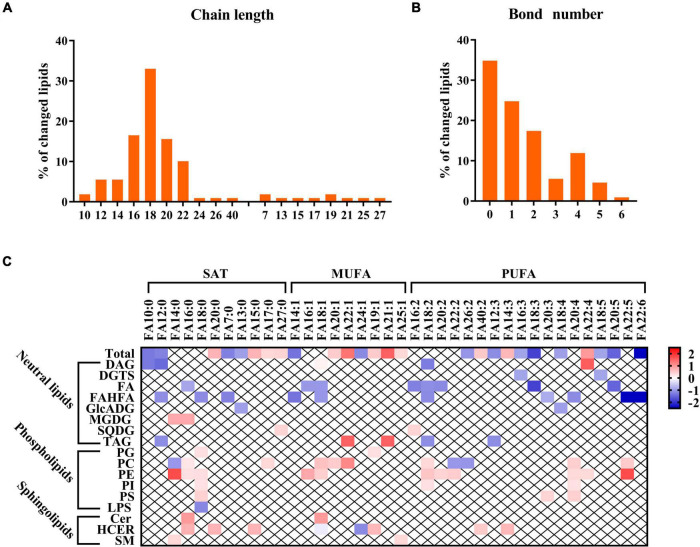
Breast milk lipids structure analysis in relation to GDM. **(A,B)** Chain length and bond number of breast milk lipids in relation to GDM. **(C)** Relationship between GDM and fatty acid composition. Red and blue colors denote lipid structure changes between GDM and NC group.

The lipidomics profiling also provides comprehensive coverage of lipid composition and configuration to help better understand the lipid species’ biochemical structure linking to GDM (20). As shown in Figure 4A, the number of carbon atoms spread over a wide range from 7 to 40, but it was obvious that most lipid chains were odd and mainly focused between 16 and 22. Moreover, we can observe that the bond number of altered lipids in human milk ranged from 0 to 6 (Figure 4B). We also performed a relationship analysis between GDM and fatty acid composition from the perspective of specific fatty acid chains in lipids. For total fatty acids, two monounsaturated fatty acids (MUFA) (FA 22:1 and FA 21:1) were strongly positively associated with GDM. In comparison, two polyunsaturated fatty acids (PUFA) (FA 18:3 and FA 22:6) were negatively associated with GDM (Figure 4C). When referring to classification, the negatively associated fatty acids mainly belonged to FA and FAHFA, while the positively associated fatty acids were PE, PI, and PS classes (Figure 4C). However, we did not identify a clear pattern in association with structures in the other dysregulated lipids.

### Breast milk lipids in relation to infant physical development

The primary outcomes for infant physical development were collected at birth, 42 days and 3 months (Table 2). The birth weight of infants born to the GDM group was significantly higher than the NC group, although these differences gradually disappeared with increasing age. The diabetic state during pregnancy led to a remarkable increase in head circumference (HC). However, no significant difference was observed between these two groups in body length. Breast milk is critical for the healthy physical growth and development of infants. Offspring born to mothers with GDM are at high risk of prematurity and macrosomia, while breastfeeding confers protection against these medical complications (21). Considering that the benefits of breastfeeding on infant’s health are due to the specific composition of breast milk, the lipid constituents in breast milk may be involved in the regulation of growth and development in the neonatal period and infancy.

**TABLE 2:** Growth and development of infants born to mothers with or without GDM.

	Weight (kg)			Body length (cm)			Head circumference (cm)		
	NC	GDM	*P*-value	NC	GDM	*P*-value	NC	GDM	*P*-value
At birth	3.34 ± 0.27	3.64 ± 0.34	**<0.001** **	49.89 ± 0.74	50.30 ± 0.86	0.12	N/A	N/A	N/A
42 days	5.01 ± 0.39	5.11 ± 0.45	0.50	55.44 ± 2.09	56.08 ± 2.00	0.34	37.40 ± 0.90	38.11 ± 1.01	0.02*
3 months	6.90 ± 0.54	6.96 ± 0.73	0.80	61.72 ± 2.78	62.45 ± 2.20	0.37	40.13 ± 1.07	41.06 ± 1.02	0.01*

Data are shown as mean ± SD. *P < 0.05, **P < 0.01 vs. NC group, unpaired t-test. The bold values mean significant difference. N/A, not applicable.

**FIGURE 5:**
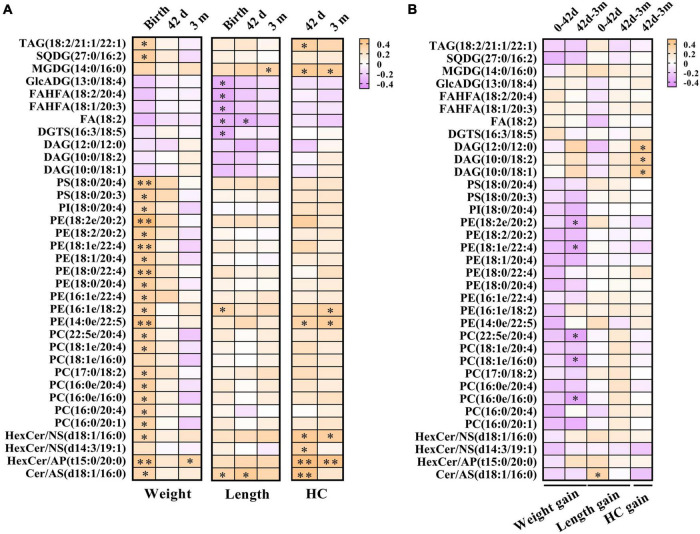
Breast milk lipids in relation to infant physical development. **(A)** Lipid species in breast milk showed remarkable association with body weight, length, and HC. **(B)** Lipid species in breast milk correlated with bodyweight gain, length elevation, and HC increase. Orange color represents positive correlation and purple indicates negative correction. *P < 0.05 and **P < 0.01. HC, head circumference.

**FIGURE 6:**
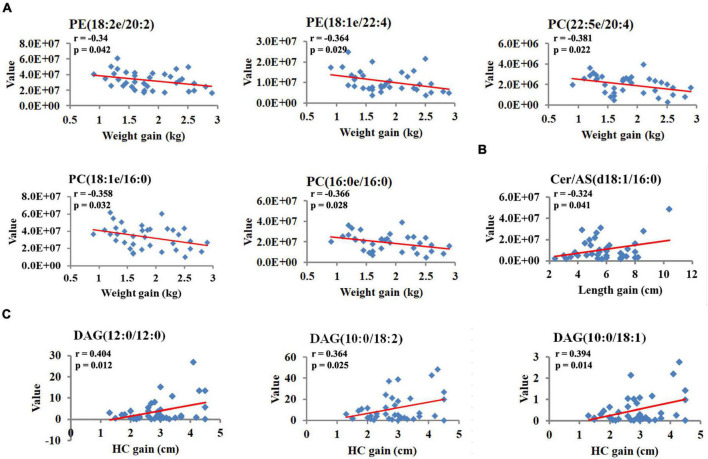
Breast milk lipids are associated with indicators of infant physical development. **(A)** The correlation coefficients between breast milk lipids and bodyweight gain. **(B)** The correlation coefficients between breast milk lipids and length gain. **(C)** The correlation coefficients between breast milk lipids and HC gain. *R* and *P*-values presented are from Pearson’s analysis.

To investigate the relationships between breast milk lipids related to GDM and infant physical development indicators, Pearson’s correlation analysis was performed. Of note, a large body of lipid species was positively associated with birthweight, whereas these correlations strikingly declined at 42 days and 3 months (Figure 5A). Moreover, neutral lipids such as GlcADG, FAHFA, FA, and DGTS revealed negative associations with the body length at birth, but rapidly diminished with increasing age (Figure 5A). We also found that sphingolipid family members (HexCer/NS, HexCer/AP, and Cer/AS) showed positive correlations with HC at 42 days, and these associations remained significant but slightly attenuated at 3 months (Figure 5A). Intriguingly, some of these lipid species showed a moderate correlation with bodyweight gain, length elevation, and HC increase, respectively, from 42 days to 3 months (Figure 5B). Specially, PE (18:2e/20:2), PE (18:1e/22:4), PC (22:5e/20:4), PC (16:0e/16:0), and PC (16:0e/16:0) were negatively correlated with bodyweight gain, while Cer/AS (d18:1/16:0) was positively correlated with length elevation (Figures 6A,B). Additionally, DAG (12:0/12:0), DAG (10:0/18:2), and DAG (10:0/18:1) were shown to have positive correlations with HC increase (Figure 6C).

## Discussion

As the most common complication during pregnancy, GDM always leads to adverse pregnancy outcomes and long-term metabolic dysfunctions in offspring, while breastfeeding may help to correct these improper developments programmed *in utero*. Milk fat is a dynamic composition among individuals and could adapt to changes in response to the maternal and fetal physiological or pathological states (22). It has been previously shown that the lipid composition of infant plasma was related to maternal milk fat composition (23). However, less is known about the lipids profile of breast milk from mothers with GDM. In this study, the lipids fluctuation in breast milk induced by GDM was comprehensively profiled using lipidomics analysis. Interestingly, our findings suggested that GDM pregnancy could result in dramatic alterations in lipid profiling of breast milk. We further revealed that breast milk lipids related to GDM showed notable associations with maternal glucose level and infant physical development.

It was well-recognized that breastfeeding is beneficial for offspring born to mothers with GDM, resulting in reduced risk for developing obesity and type 2 diabetes in future (21). The beneficial performance of breastfeeding depends on its specific composition. Lipids, making up 3–5% of milk composition, are the main energy source for infants. Except for contributing to energy content, lipids in breast milk also work as bioactive molecules to promote growth, cognitive development and the mature of nervous system of the infant (24). Thus, we compared the lipids profiling changes in breast milk from mothers suffering from GDM or not to better understand the functions provided to the newborn. The colostrum was selected in this study for the reasons listed below: 1. Colostrum is secreted in the first several postpartum days, when the mother is still at an abnormal state of glucose metabolism. 2. Colostrum packs full of nutrients and contains more fat and calories than mature milk. OPLS-DA results suggested that the lipid fingerprints within the GDM group can be obviously separated from the NC group, indicating that maternal diabetes state did remarkably affect the lipid composition of breast milk.

There are 833 lipids present in breast milk from both groups in our study. Of these lipid species, 60 showed a significant difference in response to GDM pregnancy. Among these dysregulated lipid species, almost 50% belong to PLs, including PC, PE, PI, PS, and PG, especially PE and PC. PLs are polar lipids in milk and function as the basic constituents of cell membranes. SM, PE, and PC are the predominant PLs in human and bovine milk. In human milk, PE is a source of arachidonic acid (ARA) and docosahexaenoic acid (DHA) in infants, thus playing an important role in early infant growth and development (25). In addition, PC acts as a supplier for choline in infants, while choline is required for membrane biosynthesis and plays key roles in fetal development, particularly in the brain (26). Notably, sphingolipids, another polar lipid class present in milk, are abundant in breast milk from women who suffered from GDM during pregnancy. The digestion products of sphingolipids, such as ceramides and sphingosines, are critical for the maintenance of membrane structure, protecting against gastrointestinal infection, and modulating the behavior of growth factors (27, 28). Thus, these findings contribute not only to understanding the benefits of breast milk for infants born to mother with GDM but also providing guidance for formula milk industries to produce products that suit infants’ special needs.

Accelerated infant weight gain is associated with increased abdominal adiposity, whereas slower neonatal growth reduces the risk to develop metabolic diseases, for example, cardiovascular healthy later in life (29). Infants delivered from mothers with GDM are prone to premature and overweight at birth, and we did observe strikingly high birth weight in infants from recruited GDM mother (4). However, these differences gradually vanished after 3 months postpartum, and this slowdown in bodyweight gain may help to correct the “programmed” changes in infants born to women with GDM. From the lipidomics analysis, we revealed that breast milk lipid species, including PE (18:2e/20:2), PE (18:1e/22:4), PC (22:5e/20:4), PC (16:0e/16:0), and PC (16:0e/16:0), were negatively associated with body weight gain from 42 days to 3 months after birth. In contrast, ceramide species member Cer/AS (d18:1/16:0) was positively correlated with body length gain. Ceramide has been reported to play an important role in bone resorption. The abnormality of ceramide could result in osteoblast metabolic disorder and dysfunction and finally influence bone formation (30). These results indicate that the lipid composition of breast milk could be individually changed to adapt to the specific demand of infants.

In general, this study revealed the relationship between lipid composition of breast milk and maternal GDM and further linked these changed lipids to indicators of infant physical development. To our best knowledge, this is the first report to dissect the difference in lipids in colostrum regarding GDM. However, there are still some limitations in our present study. For example, we only focused on colostrum in this study, so a more comprehensive assessment of mature milk will be performed in the future. Another limitation is that these still lack *in vivo* or *in vitro* experiments to explore the function of changed lipids. Nevertheless, our study still provides insights into the lipids profile changes of breast milk induced by GDM and may help to understand the physiological effects of breastfeeding.

## Data availability statement

The original contributions presented in this study are included in the article/Supplementary material, further inquiries can be directed to the corresponding authors.

## Ethics statement

The studies involving human participants were reviewed and approved by Human Research Ethics Committee of the Hospital with a permit number of 2020KY075. The patients/participants provided their written informed consent to participate in this study.

## Author contributions

XC and HZ conceived and designed the project. HZ processed the data and wrote the first draft of the manuscript. JZ and JX collected the milk samples and interpreted the clinical data. YZ and CC provided expertise in experimental design. XC and CS provided the funding and revised the final version of the manuscript. All authors contributed to the article and approved the submitted version.

## Supplementary material

Supplementary Figure 1 Lipid profiling changes in breast milk response to GDM. **(A,B)** Score scatter plots of PCA analysis for negative ion mode **(A)** and positive ion mode **(B)**. **(C,D)** The displacement test of OPLS-DA model in negative ion mode **(C)** and positive ion mode **(D)**.

## Abbreviations

1hPG: 1 h plasma glucose
2hPG: 2 h plasma glucose
ARA: arachidonic acid
CER: ceramide
DAG: diacylglycerol
DHA: docosahexaenoic acid
FA: fatty acids
FPG: fasting plasma glucose
GDM: gestational diabetes mellitus
HC: head circumference
HCER: hexosylceramide
HDLC: high-density lipoprotein cholesterol
LC/MS: liquid chromatography-tandem mass spectrometry
LDLC: lowdensity lipoprotein cholesterol
MAG: monoacylglycerol
MUFA: monounsaturated fatty acids
NC: normal controls
OGTT: oral glucose tolerance test
OPLS-DA: orthogonal projections to latent structures-discriminant analysis
PC: phosphatidylcholine
PCA: principal component analysis
PE: phosphatidylethanolamine
PI: phosphatidylinositol
PUFA: polyunsaturated fatty acids
QC: quality control
PL: phospholipid
SM: sphingomyelin
T2D: type 2 diabetes
TAGs: triacylglycerols
TC: total cholesterol
TG: triglyceride
VIP: variable importance in the projection.
