# Supplementary figures and images for: Influence of gestational diabetes mellitus on lipid signatures in breast milk and association with fetal physical development
Source: Front Nutr. 2022 Aug 10;9:924301. doi: 10.3389/fnut.2022.924301 (PMC9402091; doi:10.3389/fnut.2022.924301)

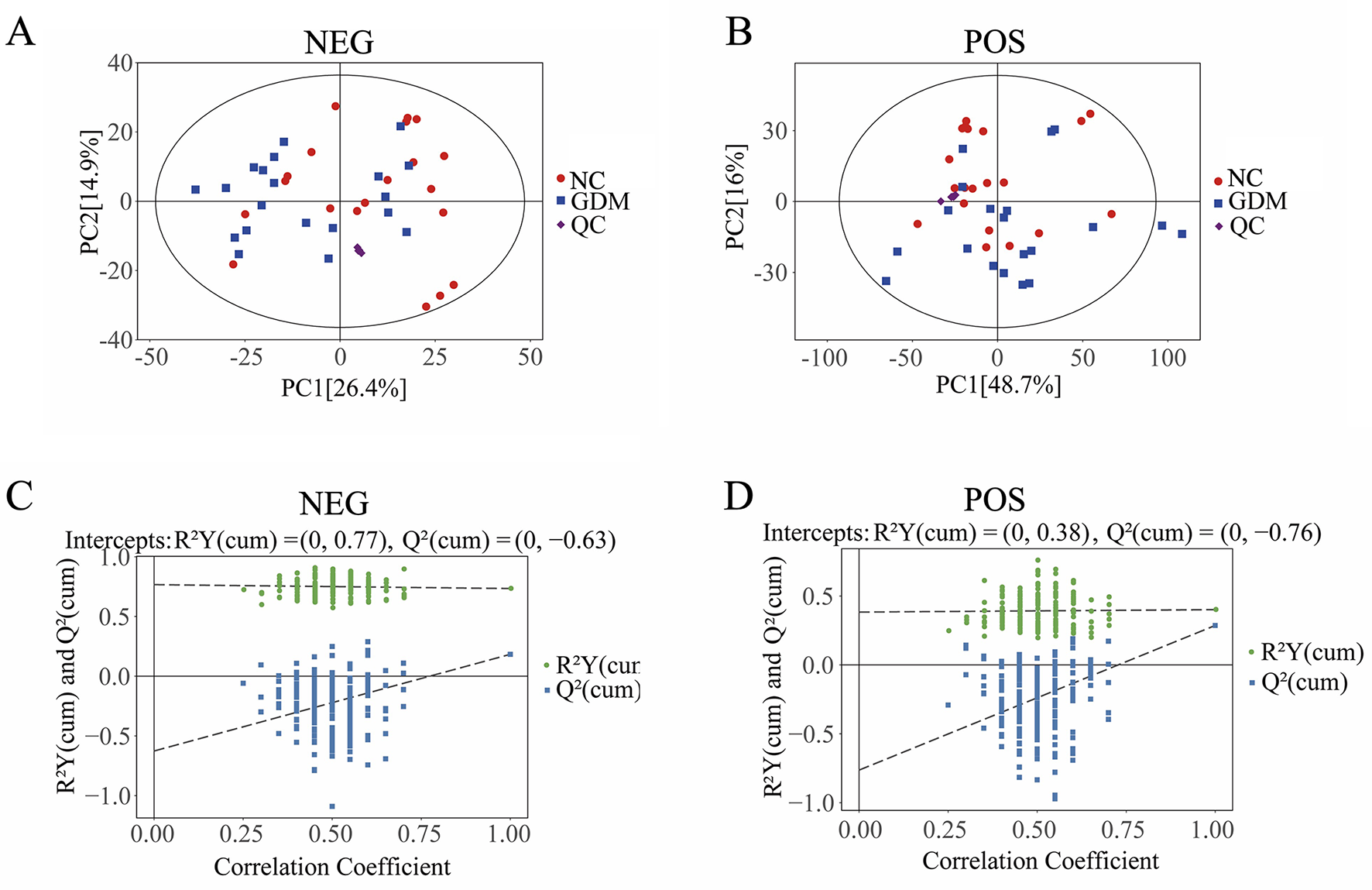

Supplement: Supplementary Figure 1 — Lipid profiling changes in breast milk response to GDM. (A,B) Score scatter plots of PCA analysis for negative ion mode (A) and positive ion mode (B). (C,D) The displacement test of OPLS-DA model in negative ion mode (C) and positive ion mode (D). [file Image_1.TIF]
